# New Journals

**Published:** 1866-01

**Authors:** 


### New Journals.

We have the following announcement of a medical journal to be published in New York, and desire to give the publishers the the opportunity of speaking for themselves, and so copy their propositions for the benefit of our readers:

"The undersigned take pleasure in announcing to the medical profession that they will begin on March 1st, 1866, the publication of a first-class medical journal, entitled The Medical Record, to be issued on the 1st and 15th of each month, and consisting of twenty-four double-column, super-royal octavo pages, printed on fine paper, with neat, clear type, forming a large handsome volume of about 600 pages at the end of each year. It will contain original articles from the first men of the country in their respective departments, reports of lectures, societies, and hospitals, an account of the progress of medical sciences throughout the world, medical news, notices of books and new instruments, editorial articles, etc., etc. It is proposed, whenever the articles require, to have them illustrated with finely engraved wood-cuts. The editorial management has been confided to Dr. George F. Shrady, of this city, whose experience in medical journalism is a guarantee that the paper will be conducted in such a manner as to receive the confidence and respect as well as meet the wants of the profession throughout every section of the country. He will be assisted by associate editors in several of the larger cities of America, e c., etc., with some of whom our arrangements are already perfected, and so soon as we can complete the list we shall publish their names. The services of able short-hand reporters have been secured for the lecture department. In a word, both editor and publisher will exert themselves to the utmost to make THE MEDICAL RECORD thoroughly worthy the countenance and patronage of the profession, useful in their practice, and interesting in their leisure moments. The specimen number, which will be the first of the volume, will be published on or about February 1st. The names of some of the leading contributors to the first volume will be announced in the first number. Terms of subscription, $4 per annum, payable in advance.

"WM. WOOD & Co., Publishers,
"61 Walker street, New York."

We have also received the following Prospectus of the ATLANTA MEDICAL AND SURGICAL JOURNAL (new series):

"After an absence of four years, the ATLANTA MEDICAL AND SURGICAL JOURNAL will make its appearance again on the first day of January, 1866. It is desirable to issue the first number at the opening of the Atlanta Medical College, on the first of November next, but the destroying hand of war has made it impracticable to attempt its publication before the middle of the session. We hope our old patrons will not only send on their names and subseription fee, but make our journal the medium of communicating such views as may be of interest to the profession. At no period since our connection with journalism has a medical periodical been so necessary as at the present time. The four years of active duty, to a large extent in military practice, without any communication with each other through tbe press, afford physicians rich stores of valuable information for the profession generally, Our object, in the conduct of the Journal will be mainly to promote advancement in the medical sciences, but we will fearlessly, honorably and indepently advocate such principles in ethics and other subjects connected with the body medical as will be most conducive to the harmony of the profession, and the cause of humanity. The Journal, containing sixty-four octavo pages of reading matter, on fine paper, will be published monthly, at five dollars a year, in advance. Address J. G. & W. E. WESTMORELAND,
Editors, Atlanta, Georgia.

